# Mendelian randomization analysis of arsenic metabolism and pulmonary function within the Hispanic Community Health Study/Study of Latinos

**DOI:** 10.1038/s41598-021-92911-8

**Published:** 2021-06-29

**Authors:** Molly Scannell Bryan, Tamar Sofer, Majid Afshar, Yasmin Mossavar-Rahmani, H. Dean Hosgood, Naresh M. Punjabi, Donglin Zeng, Martha L. Daviglus, Maria Argos

**Affiliations:** 1 Institute for Minority Health Research, University of Illinois at Chicago, Chicago, IL 60612 USA; 2 Brigham and Women’s Hospital, Boston, MA 02115 USA; 3 Harvard Medical School, Boston, MA 02115 USA; 4 University of Wisconsin-Madison, Madison, WI 53706 USA; 5 Albert Einstein College of Medicine, Bronx, NY 10461 USA; 6 Johns Hopkins University, Baltimore, MD 21205 USA; 7 University of North Carolina, Chapel Hill, Chapel Hill, NC 27599 USA; 8 School of Public Health, University of Illinois at Chicago, 1603 W. Taylor Street, MC923, Chicago, IL 60612 USA

**Keywords:** Asthma, Chronic obstructive pulmonary disease, Quantitative trait, Genetics

## Abstract

Arsenic exposure has been linked to poor pulmonary function, and inefficient arsenic metabolizers may be at increased risk. Dietary rice has recently been identified as a possible substantial route of exposure to arsenic, and it remains unknown whether it can provide a sufficient level of exposure to affect pulmonary function in inefficient metabolizers. Within 12,609 participants of HCHS/SOL, asthma diagnoses and spirometry-based measures of pulmonary function were assessed, and rice consumption was inferred from grain intake via a food frequency questionnaire. After stratifying by smoking history, the relationship between arsenic metabolism efficiency [percentages of inorganic arsenic (%iAs), monomethylarsenate (%MMA), and dimethylarsinate (%DMA) species in urine] and the measures of pulmonary function were estimated in a two-sample Mendelian randomization approach (genotype information from an Illumina HumanOmni2.5-8v1-1 array), focusing on participants with high inferred rice consumption. Among never-smoking high inferred consumers of rice (n = 1395), inefficient metabolism was associated with past asthma diagnosis and forced vital capacity below the lower limit of normal (LLN) (OR 1.40, p = 0.0212 and OR 1.42, p = 0.0072, respectively, for each percentage-point increase in %iAs; OR 1.26, p = 0.0240 and OR 1.24, p = 0.0193 for %MMA; OR 0.87, p = 0.0209 and OR 0.87, p = 0.0123 for the marker of efficient metabolism, %DMA). Among ever-smoking high inferred consumers of rice (n = 1127), inefficient metabolism was associated with peak expiratory flow below LLN (OR 1.54, p = 0.0108/percentage-point increase in %iAs, OR 1.37, p = 0.0097 for %MMA, and OR 0.83, p = 0.0093 for %DMA). Less efficient arsenic metabolism was associated with indicators of pulmonary dysfunction among those with high inferred rice consumption, suggesting that reductions in dietary arsenic could improve respiratory health.

## Introduction

Worldwide, more than 140 million people are chronically exposed to inorganic arsenic (iAs)^1^, and this exposure has been linked to both malignant and non-malignant pulmonary dysfunction^2–6^. As with many arsenic-associated diseases^7–13^, poor pulmonary outcomes are associated with an exposed individual’s ability to metabolize arsenic^11–13^. The metabolism of iAs is a multi-stage process, during which iAs can remain nmethylated, or exist in one of several methylated states^14^. In this process, the most abundant intermediate metabolite is monomethylarsenate (MMA), which can be further methylated to produce dimethylarsinate (DMA)^14^. People who are “inefficient metabolizers” are characterized by having higher relative percentages of iAs and MMA in their blood and urine, and “efficient metabolizers” are characterized by having a higher percentage of DMA. Arsenic metabolism appears to have an inherited component, as multiple genome-wide association studies have identified locations in the genome where variation is associated with an efficient arsenic metabolism phenotype^15–18^.

The health consequences of inefficient inorganic arsenic metabolism have largely been studied in populations who have been exposed to drinking water with high levels of inorganic arsenic (exceeding the 10 µg/L threshold established by the World Health Organization), or populations with known occupational exposure^7–9,12,13^. In populations with no known water or occupational exposure, arsenic was often assumed to be low, and in these populations, few studies have measured iAs metabolism. However, rice has recently been recognized as a significant route of exposure to inorganic arsenic^19,20^, suggesting that in populations with high rice consumption, arsenic exposure may reach a sufficient level such that inefficient metabolizers could be at increased risk for arsenic-associated diseases.

In study populations where inorganic arsenic metabolism﻿ has not been directly measured, but the study population has been genotyped, it is possible to study its health effects indirectly through the use of a two-sample Mendelian randomization approach^21^. This approach exploits the reported relationship between individual genotypes and the phenotype of arsenic metabolism^15–18^. Using an instrumental variable framework, these genotype-arsenic metabolism associations are then leveraged to estimate the arsenic metabolism efficiency in each genotyped study participant. This can then be combined with study-specific associations between genotype and pulmonary function. If certain assumptions are met, this estimate reflects the effect of arsenic metabolism efficiency on pulmonary traits in the target study population. As such, in populations where there may be arsenic exposure due to diet, a Mendelian randomization approach could identify whether inefficient arsenic metabolizers are at higher risk of disease.

This study examines evidence for an association between inorganic arsenic metabolism and measures of pulmonary dysfunction in a Hispanic/Latino population in the United States by implementing a two-sample Mendelian randomization approach. The study focuses on those with estimated higher inorganic arsenic exposure due to diet, as the effect of arsenic metabolism is only expected to be seen in those with appreciable arsenic exposure.

## Methods

### Study population

The Hispanic Community Health Study/Study of Latinos (HCHS/SOL) is a community-based cohort study of Hispanic and Latino adults in four cities in the United States: Chicago, Miami, the Bronx, and San Diego. The baseline examination^22^ took place between 2008 and 2011 enrolling 16,415 participants. After restricting to participants with genotyping and active consent (n = 12,633), and those without missing data (described below), 12,602 participants were included in the asthma analysis and 11,192 were included in the spirometry analysis.

### Genotyping in HCHS/SOL

The genotyping and quality control for HCHS/SOL are described elsewhere^23^. Briefly, DNA from blood was genotyped on a custom Illumina HumanOmni2.5-8v1-1 array. Samples were excluded due to sex mismatch, chromosomal anomalies, high missing call rates, and evidence of contamination or batch effects. 12,633 samples passed quality control, had complete data on genetic ancestry, and active consent. Variants were excluded for high missing call rates, Mendelian errors, duplicate-sample discordance, and deviation from ancestry-specific Hardy–Weinberg equilibrium (p < 10^−5^). This cleaned data was then imputed to the 1000 Genomes Project phase 1 reference panel^24^. SHAPEIT2 (v.2.r644)^25^ pre-phased, and IMPUTE2 (v.2.3.0)^26^ implemented the imputation.

### Identifying variants associated with arsenic metabolism efficiency

A literature search was performed through PubMed and the NHGRI-EBI GWAS catalog to identify genetic variants that influence arsenic metabolism efficiency. A variant was included if the p-value of the association was below 5 × 10^–8^, and the relationship was measured linearly as %iAs, %DMA, and %MMA. Three single nucleotide variants (SNVs) in two studies met these criteria (Table 1)^7,27^. While other variants have been identified (e.g. Refs.^17,28–31^), our approach required that the variants had their effects modeled linearly, a criteria met only by those listed in Table 1.

**Table 1:** Effect sizes and standard errors for SNV-arsenic metabolism efficiency relationships found in published literature.

rs number	chr:pos	eff/ref	%iAs		%MMA		%DMA	
			Beta	Se	Beta	Se	Beta	Se
rs9527^27^	10:104623578	T/C	1.81	0.37	2.01	0.28	− 3.82	0.48
rs11191527^27^	10:104795134	C/T	1.32	0.27	0.98	0.20	− 2.30	0.35
rs61735836^7^	21:47572887	A/G	2.71	0.37	2.42	0.29	− 5.09	0.51

*chr:pos* chromosome and position. Position locations refer to the hg19 assembly, *%iAs* percent of inorganic arsenic, *%MMA* percent of monomethylarsenate, *%DMA* percent of dimethylarsinate, *eff/ref* the effect and reference allele from the literature, *se* standard error.

Of the three identified variants, one (rs11191527) was directly measured by the HCHS/SOL genotyping array. The other two were imputed with high confidence (r^2^ = 1 for both using IMPUTE2 metrics).

### Smoking status

The analysis was stratified by ever- and never-smoking history to allow for potentially different associations by smoking history. This was done to account for the effect tobacco smoking has on lung function^32^, the differential arsenic methylation profile seen in smokers^33^, and previous research that suggests that the health impacts of arsenic may be different in smokers^34–36^. Former smokers were grouped with ever smokers rather than never smokers in order to account for the sustained reduced lung function and increased asthma risk seen in those who quit smoking^37,38^. Ever- and never-smoking history was determined by self-report from the baseline questionnaire, with never-smoking defined as smoking fewer than 100 cigarettes over a lifetime. Thirteen participants did not answer the smoking questions and were therefore excluded. 5079 ever-smokers and 7541 never-smokers remained for analysis.

### Classifying arsenic exposure through rice consumption

We ranked the study participants according to their inorganic arsenic exposure by inferring their rice consumption from answers to a food frequency questionnaire. In this study population, water is unlikely to be a source of arsenic exposure, as all participants were served by public water systems that show no evidence of arsenic contamination, with all four cities reporting arsenic concentrations below 2 µg/L or below the detectable limit^39–42^. While various foods can provide arsenic exposure, we focused on the variability in inorganic exposure through variability in rice consumption, since rice has consistently been identified as the main source of dietary iAs in studies that estimate dietary contributions of it^43,44^. Seafood can contain arsenic; however the arsenic species found in seafood is largely organic arsenobetaine, which is not metabolized, and does not have strong evidence of toxicity^45,46^. Although the level of arsenic found in rice can vary based on the location of cultivation, the harvesting method, and cooking style^47^, in the United States, most rice varieties available for purchase contain between 75 and 165 µg/kg of inorganic arsenic (along with DMA)^48^, such that among food products reported by HCHS/SOL, rice is the most commonly consumed food with high concentration of inorganic arsenic. Taken together, this suggests that characterizing rice consumption will provide an adequate ranking of participants with regard to their exposure to inorganic arsenic.

Rice consumption was inferred on the basis of two 24-h recalls of food intake, completed by HCHS/SOL participants, at enrollment and within one month of enrollment. Eleven participants did not complete the diet recall section and were excluded.

In the diet recall, the number of servings of “grains, flour, and dry mixes” that were eaten over the last day were assessed by three questions: one question focused on whole grain, the second on mixed-whole-grain, and the third on refined grains. While these three questions directed the participants to consider food in addition to rice (including amaranth, barley, buckwheat, corn flour, millet, oats, rye, sorghum, spelt, teff, triticale, quinoa, and wheat flour as well as rice), pasta and corn tortillas were assessed separately, suggesting that rice is the primary grain that participants would have referred to for this question. Further, in the subset of participants (n = 2048) who later answered a separate food propensity questionnaire, those who were above the 80th percentile in grain consumption were more than twice as likely to report eating rice more than once a week (OR 2.33, p = 1.05 × 10^–4^) and more than once a day (OR 2.69, p = 1.02 × 10^–14^), suggesting that grain consumption can be used to identify many of the high rice eaters.

In order to have a sufficient sample size for the binary outcomes assessed (statistical model described below), the top fifth of grain consumers (i.e.: cutoff at the 80th percentile) were classified as “high inferred consumers of rice.” These participants each reported more than 3.4 servings of grains (1.7 cups) over the two recalls (n = 2522 high consumers; 10,087 low consumers).

### Assessment of pulmonary function and asthma history

All participants without recent cardiovascular events or surgery were invited to perform prebronchodilator spirometry (n = 12,095 with spirometry). Spirometry was conducted in accordance with European Respiratory Society and American Thoracic Society (ATS) guidelines^49^ using a dry rolling seal spirometer with automated quality checks (Occupational Marketing, Inc., Houston, TX) with overreading by one investigator and a three-curve acceptability minimum. Participants whose effort was rated as “maximal” and whose FVC quality attribute was rated “A,” “B” or “C” were included in the spirometry analysis (11,192 (89%) participants; A = “exceeds ATS data collection standards”, B = “meets ATS data collection standards,” C = “potentially usable value, but does not meet all ATS standards”; an industry-recognized rubric that classifies whether the measurement conforms to ATS standards for a usable value^49^). In order to better model the participants who used asthma medications [n = 899 (7%) on asthma medications], their FVC and FEV_1_ were multiplied by 0.88 (the mean difference found in Du et al.,^50^ attenuated to reflect imperfect medication adherence) to estimate a participant’s spirometry result without medication.

Pulmonary dysfunction is often characterized identifying those whose spirometry measures fall below a population-based Lower Limits of Normal (LLNs). These LLNs are identified by characterizing the distribution of each spirometry measure in a healthy reference population, within strata of ethnic background, sex, age, and height. The LLN threshold is defined at the fifth percentile of each spirometry measure in this reference population, and in the wider population, those below this LLN have been found to be at higher risk of respiratory-related morbidity and mortality^51–53^. HCHS/SOL participants’ spirometry measures of FEV_1_, FVC, the ratio of FEV_1_ to FVC, and PEF were compared to the respective distributions in a healthy Hispanic/Latino reference population^54^ to identify participants below the LLN.

History of asthma was assessed through self-report from a standard questionnaire^55,56^. Participants were classified as having lifetime asthma if they answered yes to “have you ever had asthma?” and “was it diagnosed by a medical doctor?” Asthma diagnosis was further refined as “current” asthma if the participant answered yes to “do you still have asthma?” or they reported use of an anti-asthmatic medication in the last year, and “past” asthma otherwise.

### Statistical methods for SNV-pulmonary trait associations within HCHS/SOL

The relationships between each of the three SNVs and the pulmonary traits were calculated using a mixed-effect linear model^23^ using the GENESIS R package^57^. GENESIS controls for both the clustering and complex survey design utilized by HCHS/SOL, and makes use of mixed effect matrices to control for kinship, household, and block group. In addition, the top five principal components, genetically-ascertained ancestry group^58^, and the log of the sampling weights were controlled for as covariates via their inclusion as fixed effects. Each analysis was repeated within sub-strata of inferred rice consumption and smoking status.

### Statistical methods for Mendelian randomization

To estimate the arsenic metabolism-pulmonary trait relationships, the SNV-trait associations (calculated as described above) were combined with the genotype-arsenic metabolism effect sizes and standard errors (as listed in Table 1) using the process described by Burgess et al.^21^ While there are several methods for incorporating multiple SNVs into Mendelian randomization analyses^59,60^, the Burgess method mitigates the possibility of inflating type I error due to correlation between the variants, (between rs9527 and rs11191527, R^2^ = 0.28, D′ = 0.87). In this method, principal components of the three identified variants were calculated; these principal components were then used as the instrument that estimated the effect of the genetically-influenced aspects of arsenic metabolism on the respiratory traits^21^.

### Sensitivity to modeling assumptions

To assess whether medical diagnosis of asthma was sensitive to arsenic metabolism, the analysis was repeated including the 6% of asthmatics with self-reported asthma that had never been diagnosed by a doctor. To assess whether absolute levels of FEV_1_, FVC, and PEF were more sensitive to arsenic metabolism than the threshold of below LLN, each was analyzed continuously. Similarly, the percentages of FEV_1_, FVC, and PEF as a fraction of each’s predicted values were also analyzed continuously to determine whether those phenotypes were more sensitive to arsenic metabolism than the dichotomous outcome of being below the LLN. To evaluate whether the spirometry results were sensitive to the correction for medication use, the spirometry analyses were repeated on uncorrected data where medication use was controlled for as a confounder, and again in data where those on medications were excluded. To assess whether the results were sensitive to the quality of the spirometry measurements, the analysis was repeated excluding results where the quality of the FEV_1_ and FVC curves were rated “C”, which did not fully meet the American Thoracic Society standards, but was still rated as “potentially usable”.

### Ethics approval and consent to participant

The study was conducted with the approval and oversight of the Ethics and Institutional Review Boards of Albert Einstein School of Medicine, University of Illinois Chicago (2013-1261), University of Miami, and San Diego State University, as well as the coordinating center institutions University of North Carolina Chapel Hill and University of Washington. Informed consent was obtained from all participants. All methods were performed in accordance with the relevant guidelines and regulations.

## Results

Characteristics of the study population are found in Table 2. The Mendelian randomization estimates of the effect of arsenic metabolism and pulmonary function for high inferred consumers of rice are presented in Table 3, stratified by smoking status.

**Table 2:** Characteristics of the study sample.

	High inferred rice consumption	Low inferred rice consumption
	n = 2522	n = 10,087
**Gender**		
Male	1485 (58.9%)	3686 (36.5%)
Female	1037 (41.1%)	6401 (63.5%)
Mean age in years (SD)	45 (14)	46 (14)
**Enrollment site**		
Bronx	502 (19.9%)	2711 (26.9%)
Chicago	491 (19.5%)	2572 (25.5%)
Miami	1233 (48.9%)	2197 (21.8%)
San Diego	296 (11.7%)	2607 (25.8%)
**Genetic ancestry**		
Mexican	516 (20.5%)	4148 (41.1%)
Central American	316 (12.5%)	1064 (10.5%)
Dominican	178 (7.1%)	991 (9.8%)
Puerto Rican	394 (15.6%)	1837 (18.2%)
Cuban	860 (34.1%)	1389 (13.8%)
South American	258 (10.2%)	658 (6.5%)
**Smoking status**		
Never	1395 (55.3%)	6140 (60.9%)
Former	514 (20.4%)	2011 (19.9%)
Current	613 (24.3%)	1936 (19.2%)
**Asthma history**		
No history	2076 (82.3%)	8339 (82.7%)
Asthma in past	218 (8.6%)	679 (6.7%)
Current asthma	173 (6.9%)	862 (8.5%)
Missing	55 (2.2%)	207 (2.1%)
**Asthmatic medication use**		
Yes	140 (5.6%)	759 (7.5%)
Mean FEV_1_, mL (SD)	3078 (835)	2801 (785)
Mean FVC, mL (SD)	3847 (980)	3490 (935)
Mean PEF, mL/s(SD)	7922 (2061)	7217 (1938)
Mean FEV_1_/FVC, percent (SD)	80 (7)	80 (7)

*SD* standard deviation, *FEV*_*1*_ forced expiratory volume in one second, *FVC* forced vital capacity, *PEF* peak expiratory flow.

**Table 3:** Mendelian randomization estimates for the associations between three measures of arsenic metabolism efficiency and asthma-associated traits among those with high inferred rice consumption (n = 2522).

Metabolite	Ever-smokers		Never-smokers		
	Odds ratio (95% CI)	p-value	Odds ratio (95% CI)		p-value
	n = 1127		n = 1395		
**Lifetime asthma**					
%iAs	1.05 (0.82–1.34)	0.7152	1.13 (0.90–1.41)		0.2862
%MMA	1.05 (0.88–1.25)	0.5896	1.09 (0.93–1.28)		0.2811
%DMA	0.98 (0.88–1.08)	0.6298	0.95 (0.86–1.04)		0.2793
**Current asthma**					
%iAs	1.10 (0.79–1.55)	0.5718	0.88 (0.64–1.20)		0.4062
%MMA	1.07 (0.84–1.37)	0.5583	0.91 (0.71–1.15)		0.4231
%DMA	0.96 (0.83–1.10)	0.5484	1.06 (0.92–1.22)		0.4002
**Past asthma**					
%iAs	0.98 (0.71–1.35)	0.9123	1.40 (1.05–1.86)		0.0212*
%MMA	1.02 (0.81–1.28)	0.8549	1.26 (1.03–1.54)		0.0240*
%DMA	1.00 (0.87–1.14)	0.9492	0.87 (0.77–0.98)		0.0209*
**FEV1 below LLN**					
%iAs	1.06 (0.83–1.37)	0.6225	1.24 (0.97–1.60)		0.0862
%MMA	1.04 (0.87–1.24)	0.6827	1.10 (0.92–1.31)		0.3139
%DMA	0.98 (0.88–1.08)	0.6462	0.93 (0.84–1.04)		0.1962
**FVC below LLN**					
%iAs	1.09 (0.83–1.42)	0.5299	1.42 (1.10–1.83)		0.0072*
%MMA	1.05 (0.87–1.27)	0.6013	1.24 (1.03–1.50)		0.0193*
%DMA	0.97 (0.87–1.08)	0.5621	0.87 (0.78–0.97)		0.0123*
**FEV1/FVC below LLN**					
%iAs	1.19 (0.90–1.58)	0.2099	1.00 (0.70–1.44)		0.9845
%MMA	1.11 (0.91–1.35)	0.3003	0.98 (0.75–1.27)		0.8739
%DMA	0.94 (0.83–1.05)	0.2575	1.01 (0.86–1.17)		0.9177
**PEF below LLN**					
%iAs	1.54 (1.10–2.15)	0.0108*	1.00 (0.68–1.46)		0.9955
%MMA	1.37 (1.08–1.73)	0.0097*	0.97 (0.73–1.27)		0.8122
%DMA	0.83 (0.72–0.96)	0.0093*	1.01 (0.86–1.19)		0.8849

High inferred consumers of rice are those above the 80th percentile of consumption for grains.

The reported coefficients are interpreted as the expected increase in the odds of the trait for a one percentage point increase in the arsenic metabolite.

*LLN* Lower limit of normal, *FEV*_*1*_ forced expiratory volume in one second (mL), *FVC* forced vital capacity (mL), *PEF* peak expiratory flow (mL/s), *%iAs* percent of inorganic arsenic, *%MMA* percent of monomethylarsenate, *%DMA* percent of dimethylarsinate.

### Never smokers with high inferred rice consumption

Among never-smokers (Table 3, right), inefficient metabolism was associated with an increased risk of a past asthma diagnosis (OR 1.40, 95% CI 1.05–1.86 for %iAs; OR 1.26, 95% CI 1.03–1.54 for %MMA; OR 0.87, 95% CI 0.77–0.98 for %DMA). Additionally, FVC below the LLN was associated with inefficient arsenic metabolism (OR 1.42 95% CI 1.10–1.83 for %iAs; OR 1.24, 95% CI 1.03–1.50 for %MMA; OR 0.87, 95% CI 0.78–0.97 for %DMA). There was no strong evidence for association with any of the other tested spirometry comparisons to LLN.

### Ever smokers with high inferred rice consumption

Among ever-smokers (Table 3, left), inefficient arsenic metabolizers were more likely to have a PEF below the LLN, as for each percentage-point increase in iAs, there was a 54% higher odds of PEF below LLN (OR 1.54, 95% CI 1.10–2.15). Similar detrimental effects on PEF were found with each percentage-point increase in MMA (OR 1.37, 95% CI 1.08–1.73). For each percentage point increase in DMA (the marker of efficient arsenic metabolism), there was a 17% decrease in the odds that a participant would have PEF below LLN (OR 0.83, 95% CI 0.72–0.96). There was no strong evidence for association with asthma or any of the other tested spirometry comparisons to LLN.

### Low inferred rice consumption

Among intermediate- and low-consumers of grain, while the magnitude of the associations is often consistent with inefficient arsenic metabolism being associated with pulmonary function, the confidence intervals are generally wide. Given the borderline significance of the two tests that passed the significance threshold, there was no convincing pattern between arsenic metabolism and pulmonary function for either ever-smokers (Table 4, left) or never-smokers (Table 4, right).

**Table 4:** Mendelian randomization estimates for the associations between three measures of arsenic metabolism efficiency and asthma-associated traits among those with low inferred rice consumption (n = 10,087).

Metabolite	Ever-smokers		Never-smokers	
	Odds ratio (95%CI)	p-value	Odds ratio (95%CI)	p-value
	n = 3947		n = 6140	
**Lifetime asthma**				
%iAs	1.07 (0.95–1.21)	0.2754	1.14 (0.92–1.41)	0.2120
%MMA	1.04 (0.95–1.14)	0.3960	1.10 (0.94–1.28)	0.2294
%DMA	0.97 (0.92–1.03)	0.3374	0.95 (0.86–1.04)	0.2215
**Current asthma**				
%iAs	1.09 (0.93–1.28)	0.2858	0.90 (0.66–1.21)	0.4808
%MMA	1.06 (0.94–1.19)	0.3510	0.92 (0.73–1.16)	0.4924
%DMA	0.97 (0.90–1.03)	0.3152	1.05 (0.92–1.20)	0.4674
**Past asthma**				
%iAs	1.07 (0.90–1.28)	0.4509	1.06 (0.95–1.18)	0.3037
%MMA	1.03 (0.91–1.18)	0.6270	1.05 (0.97–1.14)	0.2178
%DMA	0.98 (0.90–1.06)	0.5512	0.97 (0.93–1.02)	0.2461
**FEV1 below LLN**				
%iAs	1.13 (0.99–1.28)	0.0617	0.96 (0.86–1.08)	0.4976
%MMA	1.10 (1.01–1.21)	0.0367*	0.98 (0.90–1.07)	0.6839
%DMA	0.95 (0.90–1.00)	0.0414*	1.01 (0.96–1.07)	0.6026
**FVC below LLN**				
%iAs	0.98 (0.85–1.11)	0.7103	0.99 (0.88–1.11)	0.8596
%MMA	0.98 (0.89–1.08)	0.6520	1.00 (0.92–1.09)	0.9822
%DMA	1.01 (0.96–1.07)	0.6773	1.00 (0.95–1.05)	0.9256
**FEV1/FVC below LLN**				
%iAs	1.13 (0.97–1.32)	0.1057	0.89 (0.75–1.05)	0.1707
%MMA	1.08 (0.97–1.21)	0.1515	0.93 (0.82–1.06)	0.2825
%DMA	0.95 (0.89–1.01)	0.1246	1.05 (0.97–1.13)	0.2388
**PEF below LLN**				
%iAs	0.98 (0.83–1.16)	0.8151	1.04 (0.88–1.24)	0.6296
%MMA	1.02 (0.90–1.15)	0.8038	1.01 (0.89–1.14)	0.9350
%DMA	1.00 (0.93–1.07)	0.9527	0.99 (0.92–1.07)	0.8244

Low inferred consumers of rice are those below the 80th percentile of consumption for grains.

The reported coefficients are interpreted as the expected increase in the odds of the trait for a one percentage point increase in the arsenic metabolite.

*LLN* Lower limit of normal, *FEV*_*1*_ forced expiratory volume in one second (mL), *FVC* forced vital capacity (mL), *PEF* peak expiratory flow (mL/s), *%iAs* percent of inorganic arsenic, *%MMA* percent of monomethylarsenate, *%DMA* percent of dimethylarsinate.

### Sensitivity analyses

Broadening the definition of asthma history to include those who reported a history of asthma but did not receive a diagnosis leads to substantively similar results (Supplementary Table 1). Modeling the spirometry measures directly or as a percentage of the predicted value for their ethnicity, age, height, and gender were both less sensitive to arsenic metabolism than modeling whether the participant had passed the clinically relevant threshold of below the lower limit of normal (Supplementary Table 2), although the point estimates are consistent with a protective effect of efficient metabolism and a detrimental effect of inefficient metabolism.

The results were not sensitive to the method used to control for medication, or the quality of spirometry measures included in the analysis (tables available upon request).

## Discussion

This analysis suggests that inefficient metabolism of inorganic arsenic is associated with a history of asthma and signs of pulmonary dysfunction. Further, we find that these effects were observed at levels of arsenic exposure that could be acquired through diets that are high in rice. The pulmonary traits that were most influenced by arsenic metabolism differed by smoking history. For never-smokers, inefficient metabolism was associated with increased odds of a past history of asthma and FVC being below the LLN. For each percentage-point increase in %iAs, the odds of a past history of asthma increased by more than 40%. A similar magnitude of effect was seen on the odds of FVC dropping below the LLN. Among ever-smokers, PEF was the most responsive spirometry measure to inefficient arsenic metabolism, with a percentage point increase in %iAs associated with a 60% increase in the odds that the participant’s PEF fell below LLN. There were similar detrimental effects seen for increases in %MMA, and protective effects for decreases in %DMA. Given that the risk alleles for inefficient metabolism were each associated with an increase in %iAs between 1.3 and 2.7 percentage points (Table 1), this suggests that arsenic metabolism may be responsible for significant variability in pulmonary function.

As we did not expect genotype to increase risk in the absence of arsenic, those participants whose inferred rice consumption was low served as a negative control. In this population a less-clear pattern emerged connecting the ability to methylate arsenic and pulmonary dysfunction (Table 4).

Our analysis utilized a Mendelian randomization approach to complement the existing literature that has suggested that arsenic affects pulmonary function at higher levels of exposure^11–13^. Our work builds upon this earlier research by providing additional evidence to support the hypothesis that at levels of exposure that are consistent with high rice consumption but no known water exposure, poor arsenic metabolizers may be at risk of pulmonary dysfunction, and we also find that FVC may be particularly sensitive to this effect^4^. Although our analysis focused on methylation, our findings are consistent with results from the MESA study, which suggest that spirometry-based measures of lung function may be worse in participants who were daily rice eaters^61^, and those found in the Strong Heart Study, which found that respiratory dysfunction is associated with even low-to-moderate arsenic exposure^62^. Our analysis reached a different conclusion than an analysis of the 2003–2006 National Health and Nutrition Examination Survey (NHANES), which found no relationship between inorganic arsenic exposure and diagnoses of multiple respiratory diseases^63^, and a later NHANES analysis that included spirometry^64^. However, the absolute level of inorganic arsenic exposure in the NHANES population was likely lower than what would be expected in populations with high rice consumption, and these analyses looked at absolute level of arsenic, rather than metabolism efficiency.

Further work is needed to understand why some respiratory phenotypes appear to be more sensitive to arsenic metabolism than others, and whether additional phenotypes, such as control of asthma among asthmatics, may also be affected. The association between past, but not current asthma diagnosis may reflect an increased sensitivity to arsenic toxicity in childhood^36,65,66^, but additional study is needed to clarify the association. Our observation that the association between arsenic metabolism and pulmonary function differs by smoking history is consistent with other research that has found differential health effects by smoking status^36,67^. Future studies that may clarify this potential interaction could help to identify those at greatest risk of pulmonary dysfunction.

The mechanisms that underpin this effect are not yet fully understood. In vitro studies have shown arsenic to increase oxidative stress in lung cells^68^, and also induce epigenetic changes in lung tissue^69^. Animal studies have observed accumulation of arsenic metabolites in lung tissues^70^, and markers of immune dysregulation^71^ and oxidative stress^72^ in the lungs of chronically exposed mice. In human models, markers of pulmonary inflammation were elevated in the sputum of exposed individuals^73^, as well as CC16, a marker of early lung damage^5^. Taken together, this suggests several mechanisms through which arsenic exposure, and arsenic metabolism can induce respiratory dysfunction, and additional investigations into these underlying pathways are warranted.

### Mendelian randomization assumptions

The ability of our findings to reflect the influence of iAs metabolism depends on assumptions required of all Mendelian randomization and instrumental variable analyses, discussed below. While not fully testable, several lines of evidence suggest that the assumptions are valid.

1. the SNVs used in the Mendelian randomization are associated with arsenic metabolism.
  Since arsenic metabolites were not measured in HCHS/SOL, the first assumption cannot be directly tested. However, its plausibility is supported first by the multiple study populations in which variation near the *AS3MT* region has convincingly been associated with iAs metabolism^15,17,18,74,75^. Additionally, the SNVs selected for the instrument belong to biological pathways that are known to be involved in arsenic metabolism efficiency. rs9527 and rs11191527 are located in the region of the gene *AS3MT*, and rs61735836 creates a valine to methionine missense mutation in *FTCD*. Both genes encode enzymes (arsenite methyltransferase and formimidoyltransferase cyclodeaminase) involved in arsenic methylation^14,76^.
2. The SNVs-pulmonary function associations are not confounded by an unmeasured factor.
  The most common threat to this assumption is through uncontrolled population stratification in which SNVs would spuriously appear to be associated with pulmonary function due to non-genetically-influenced clustering of traits in people of similar genetic backgrounds. However, the methodology used to calculate the SNV-pulmonary function relationships within HCHS/SOL used an extensively validated algorithm which has demonstrated no inflation in type I error for multiple phenotypes within HCHS/SOL^77^. Our analysis controls for cryptic relatedness, sample clustering, and complex survey design through the mixed effects, as well as ancestral background groups, and principal components through fixed effects^23,58^.
3. The SNVs only affect pulmonary function through their effects on arsenic metabolism.

The ability to test this assumption is limited by our still-expanding knowledge of the effects of the genome. However, its plausibility is supported by the NHGRI-EBI GWAS catalog^78^, which lists no SNVs associated with any respiratory-related trait in high linkage disequilibrium (r^2^ > 0.3) with the three SNVs used in the instrument. This suggests that there are no other mechanisms through which these variants might affect respiratory function except through their influence on arsenic metabolism. While an assessment of the participants of the UKBiobank (UKBB) found a possible association between the *AS3MT* SNVs in our instrument and smoking status (http://www.phenoscanner.medschl.cam.ac.uk), the SNV-smoking effect sizes reported in the UKBB are small in magnitude and inconsistent in direction, and there is no association between the *AS3MT* SNVs and smoking in HCHS/SOL (p > 0.25 for both, with ORs close to 1), suggesting that the relationship seen in the UKBB is not responsible for the relationship observed in this analysis. Additionally, our decision to stratify by smoking status alleviates the concern that the variants act through influencing smoking status and downstream pulmonary function.

The results of this study could be strengthened if additional data were available to refine our classification of arsenic exposure in HCHS/SOL beyond the 24-h food recall of grain consumption. However, the public water systems of the HCHS/SOL participants show no evidence of elevated arsenic contamination^79–82^, so it is likely that dietary arsenic was a main source of exposure for most participants. While there is potential for misclassification, in that some high consumers of grain may have not eaten much rice, neither non-dietary routes of arsenic exposure, nor misreporting of food intake is likely to be confounded with genetics or respiratory dysfunction, and as such would only serve to introduce people into the analytic sample with low level of arsenic exposure, which would dilute our ability to estimate the effect of metabolism, but not bias the estimates.

As this analysis is one of the first to directly look at the effect of arsenic methylation capacity on pulmonary outcomes in a population with low-to-moderate exposure, we undertook several sensitivity analyses, and broadly tested multiple respiratory phenotypes, and these multiple tests increase the possibility of type I error. However, the positive associations coherently form a pattern that implicates inefficient arsenic metabolism as a risk factor for a broad range of respiratory outcomes, each of which have their own biological plausibility, and passed multiple sensitivity analyses.

In conclusion, those who inefficiently metabolize arsenic show an association with increased risk of measures of pulmonary dysfunction that are used in routine clinical practice, and our analysis suggests that dietary rice may provide enough arsenic exposure to observe this relationship. This suggests that arsenic metabolism may be a previously unappreciated risk factor for pulmonary dysfunction in the Hispanic/Latino community and in other populations in which rice is a dietary staple. These findings suggest that in addition to water-based arsenic exposure, diet should be considered as a possible route by which a participant may be within a level of exposure that can influence respiratory function, while also suggesting that a mitigation strategy aimed at rice could help to reduce the burden of respiratory dysfunction in inefficient arsenic metabolizers.

## Supplementary Information

Supplementary Information.

## Data Availability

The datasets analyzed in this manuscript are available through the completion and approval of a data access request to the Hispanic Community Health Study/Study of Latinos (https://sites.cscc.unc.edu/hchs/).
