# Supplementary material for: Mendelian randomization analysis of arsenic metabolism and pulmonary function within the Hispanic Community Health Study/Study of Latinos
Source: Sci Rep. 2021 Jun 29;11:13470. doi: 10.1038/s41598-021-92911-8 (PMC8242019; doi:10.1038/s41598-021-92911-8)
Supplement: Supplementary file 1 — Supplementary Information. [file 41598_2021_92911_MOESM1_ESM.docx]

# supplemental materials

## Title

Arsenic metabolism and pulmonary function: a Mendelian randomization analysis within the Hispanic Community Health Study/Study of Latinos

## Authors

^1^Molly Scannell Bryan

^2,3^Tamar Sofer

^4^Majid Afshar

^5^Yasmin Mossavar-Rahmani

^5^H. Dean Hosgood

^6^Naresh M. Punjabi

^7^Donglin Zeng

^1^Martha L. Daviglus

^8^Maria Argos *

## Affiliations

^1^University of Illinois at Chicago, Institute for Minority Health Research; Chicago, IL 60612, United States

^2^Brigham and Women’s Hospital; Boston, MA 02115, United States

^3^Harvard Medical School; Boston, MA 02115, United States

^4^Loyola University Medical Center; Maywood, IL 60153, United States

^5^Albert Einstein College of Medicine; Bronx, NY 10461, United States

^6^Johns Hopkins University; Baltimore, MD 21205, United States

^7^University of North Carolina, Chapel Hill; Chapel Hill, NC 27599, United States

^8^University of Illinois at Chicago, School of Public Health; Chicago, IL 60612, United States

## Corresponding Author

Maria Argos

1603 W. Taylor Street, MC923

Chicago, IL 60612

United States

+1-312-355-1584

fax: +1-312-996-0064

argos@uic.edu

## Supplemental Table 1: Mendelian randomization estimates for the associations between three measures of arsenic metabolism efficiency and asthma history with or without doctor diagnosis, for high consumers of rice

|  | | **Ever-Smoker** | | | | **Never-Smoker** | | |  |
| --- | --- | --- | --- | --- | --- | --- | --- | --- | --- |
|  | | **n=1127** | |  | | **n=1395** | |  |  |
| **Metabolite** | | **Beta (95% CI)** | | **p-value** | | **Beta (95% CI)** | | **p-value** |  |
| Ever Asthma |  | |  | |  | |  | | |
| %iAs | 1.04 (0.82 to 1.33) | | 0.7237 | | 1.14 (0.92 to 1.41) | | 0.2120 | | |
| %MMA | 1.04 (0.88 to 1.24) | | 0.6242 | | 1.10 (0.94 to 1.28) | | 0.2294 | | |
| %DMA | 0.98 (0.88 to 1.08) | | 0.6577 | | 0.95 (0.86 to 1.04) | | 0.2215 | | |
| Current Asthma |  | |  | |  | |  | | |
| %iAs | 1.12 (0.80 to 1.56) | | 0.5139 | | 0.90 (0.66 to 1.21) | | 0.4808 | | |
| %MMA | 1.07 (0.85 to 1.36) | | 0.5513 | | 0.92 (0.73 to 1.16) | | 0.4924 | | |
| %DMA | 0.96 (0.83 to 1.10) | | 0.5253 | | 1.05 (0.92 to 1.20) | | 0.4674 | | |
| Past Asthma |  | |  | |  | |  | | |
| %iAs | 0.97 (0.71 to 1.33) | | 0.8507 | | 1.39 (1.06 to 1.82) | | 0.0161* | | |
| %MMA | 1.01 (0.81 to 1.26) | | 0.9091 | | 1.26 (1.03 to 1.53) | | 0.0208* | | |
| %DMA | 1.00 (0.88 to 1.14) | | 0.9931 | | 0.87 (0.78 to 0.98) | | 0.0174* | | |

High consumers of rice are those above the 80^th^ percentile of consumption for grains

The reported coefficients are interpreted as the expected increase in the trait for a one percentage point increase in the arsenic metabolite

%iAs: percent of inorganic arsenic

%MMA: percent of monomethylarsenate

%DMA: percent of dimethylarsinate

## Supplemental Table 2: Mendelian randomization estimates for the associations between three measures of arsenic metabolism efficiency and continuous spirometry measures, for high consumers of rice

|  | **Ever-Smoker** | | **Never-Smoker** | |
| --- | --- | --- | --- | --- |
|  | **n=1127** |  | **n=1395** |  |
| **Metabolite** | **Beta (95% CI)** | **p-value** | **Beta (95% CI)** | **p-value** |
| FEV1 |  |  |  |  |
| %iAs | -24 (-97 to 49) | 0.5143 | -0 (-70 to 69) | 0.9903 |
| %MMA | -24 (-75 to 28) | 0.3590 | 7 (-41 to 55) | 0.7665 |
| %DMA | 13 (-18 to 44) | 0.3988 | -3 (-31 to 26) | 0.8614 |
| FVC |  |  |  |  |
| %iAs | -16 (-103 to 71) | 0.7142 | 3 (-80 to 86) | 0.9469 |
| %MMA | -23 (-85 to 38) | 0.4502 | 9 (-48 to 66) | 0.7631 |
| %DMA | 11 (-25 to 48) | 0.5320 | -3 (-38 to 31) | 0.8393 |
| FEV1/FVC |  |  |  |  |
| %iAs | -0.22 (-0.22 to -0.21) | 0.5470 | 0.01 (0.01 to 0.02) | 0.9589 |
| %MMA | -0.07 (-0.08 to -0.07) | 0.7678 | 0.05 (0.05 to 0.06) | 0.7707 |
| %DMA | 0.06 (0.06 to 0.07) | 0.6715 | -0.03 (-0.03 to -0.02) | 0.8240 |
| PEF |  |  |  |  |
| %iAs | -126 (-311 to 59) | 0.1782 | 104 (-72 to 280) | 0.2414 |
| %MMA | -97 (-226 to 31) | 0.1345 | 86 (-36 to 208) | 0.1615 |
| %DMA | 57 (-20 to 134) | 0.1398 | -48 (-121 to 24) | 0.1879 |
| FEV1/FEV1(predicted) |  |  |  |  |
| %iAs | -0.37 (-0.38 to -0.35) | 0.6060 | -0.63 (-0.64 to -0.62) | 0.3200 |
| %MMA | -0.08 (-0.09 to -0.07) | 0.8789 | -0.19 (-0.19 to -0.18) | 0.6649 |
| %DMA | 0.09 (0.08 to 0.09) | 0.7657 | 0.17 (0.16 to 0.17) | 0.5188 |
| FVC/FVC(predicted) |  |  |  |  |
| %iAs | -0.07 (-0.09 to -0.06) | 0.9097 | -0.80 (-0.81 to -0.79) | 0.1744 |
| %MMA | 0.00 (-0.00 to 0.01) | 0.9913 | -0.37 (-0.38 to -0.36) | 0.3569 |
| %DMA | 0.01 (0.00 to 0.01) | 0.9770 | 0.26 (0.26 to 0.27) | 0.2713 |
| (FEV1/FVC)/(predicted) |  |  |  |  |
| %iAs | -0.40 (-0.40 to -0.39) | 0.3318 | 0.33 (0.32 to 0.34) | 0.2847 |
| %MMA | -0.15 (-0.15 to -0.14) | 0.6022 | 0.28 (0.28 to 0.29) | 0.1775 |
| %DMA | 0.12 (0.12 to 0.12) | 0.4774 | -0.16 (-0.16 to -0.16) | 0.2011 |
| PEF/PEF(predicted) |  |  |  |  |
| %iAs | -0.50 (-0.52 to -0.48) | 0.5783 | 0.27 (0.26 to 0.29) | 0.7272 |
| %MMA | -0.16 (-0.17 to -0.15) | 0.7953 | 0.28 (0.27 to 0.29) | 0.6090 |
| %DMA | 0.14 (0.13 to 0.15) | 0.7103 | -0.15 (-0.15 to -0.14) | 0.6490 |

High consumers of rice are those above the 80^th^ percentile of consumption for grains

The reported coefficients are interpreted as the expected increase in the odds of the trait for a one percentage point increase in the arsenic metabolite

LLN: Lower limit of normal

FEV_1_=forced expiratory volume in one second (mL)

FVC=forced vital capacity (mL)

PEF=peak expiratory flow (mL/s)

%iAs: percent of inorganic arsenic

%MMA: percent of monomethylarsenate

%DMA: percent of dimethylarsinate
